# Advances in Analytical Determination Methods and Toxicity and Health Risk Assessment of 6PPD and Its Transformation Products in Food

**DOI:** 10.3390/toxics13121076

**Published:** 2025-12-14

**Authors:** Bolin Liu, Yu Liu, Ziyue Zhan, Ji’an Xie, Gang Ding, Ziwei Zhao

**Affiliations:** 1 Anhui Provincial Center for Disease Control and Prevention, Hefei 230601, Chinadinggang801@163.com (G.D.); 2 School of Education and Psychology, Hefei Normal University, Hefei 230601, China

**Keywords:** 6PPD, 6PPD-Q, health risks, sample pretreatment method, detection techniques

## Abstract

*N*-(1,3-Dimethylbutyl)-*N*’-phenyl-*p*-phenylenediamine (6PPD) is a member of the *p*-phenylenediamines (PPDs), recognized as a highly effective antioxidant. It has been extensively employed in the automotive tire manufacturing industry, and plays a critical role in enhancing the durability and service life of rubber materials. In recent years, significant research has demonstrated that 6PPD-quinone (6PPD-Q), the transformation product of 6PPD, is a toxic substance that causes the acute death of coho salmon (*Oncorhynchus kisutch*). The toxicity of its aquatic organisms has attracted great attention of scholars, and 6PPD-Q has been regarded as the emerging contaminant. It has been reported that 6PPD diffuses from rubber debris into environmental media such as air, soil, and water after the tires wear. 6PPD and 6PPD-Q have been widespread in the environment, and they migrate into food through the environment and enter the human body through exposure routes such as dietary intake and drinking water, posing potential risks to human health. This paper reviewed the current reports on the toxicity and health risks of 6PPD and 6PPD-Q, and compares the advantages and disadvantages of sample pretreatment methods and detection technologies of 6PPD and 6PPD-Q in different food matrices, and provides a scientific basis for food safety risk assessment. Evidence indicated that 6PPD-Q exhibits not only acute aquatic toxicity but also cytotoxicity, hepatotoxicity, neurotoxicity, and genotoxicity. Epidemiological data suggest a significant association between elevated 6PPD-Q levels and increased risks of colorectal cancer and liver abnormalities. There remains an urgent need to develop comprehensive, standardized, and high-throughput analytical methodologies for the efficient screening of 6PPD and 6PPD-Q in food samples, along with expanded dietary exposure assessments, to fully characterize the impacts of 6PPD and 6PPD-Q on human health.

## 1. Introduction

*p*-phenylenediamines (PPDs) are a class of antioxidants widely used in the rubber industry, which play a critical role in preventing thermal oxidative aging and extending the service life of rubber products [1]. *N*-(1,3-Dimethylbutyl)-*N*’-phenyl-*p*-phenylenediamine (6PPD), as a member of the PPDs family, has outstanding advantages in antioxidant properties and is widely used as an antioxidant for tires [2,3,4,5]. The presence of long-chain alkyl groups in 6PPD not only significantly enhances its antioxidant efficiency but also improves its solubility and dispersion within the rubber matrix. Additionally, the aromatic substituent contributes to increased molecular durability and chemical stability (structural formulas are shown in Table S1).Adding 6PPD to tires can prevent hardening and cracking, which can reduce durability and operational safety. In 2020, the output of 6PPD in China was 198,000 tons, accounting for 53.95% of the total output of antioxidant. Besides China, 6PPD is also the most widely used *p*-phenylenediamine antioxidant in the world [6,7]. A total of 3.1 billion tires are produced worldwide each year [8]. As tires wear particles (TWPs), generated by the friction between the tires and the road surface, accumulate in dust and airborne particles on highways and streets, 6PPD in tires was released into the environment along with TWPs. It was reported that, in Europe, the total amount of 6PPD released into the environment by rubber tires was about 130 tons per year [9]. A groundbreaking study in 2020 confirmed that the transformation products of 6PPD were toxic compounds that cause acute deaths of large numbers of coho salmon (*Oncorhynchus kisutch*) in the northwestern United States [8]. The transformation products were *N*-(1,3-Dimethylbutyl)-*N*’-phenyl-*p*-phenylenediamine-quinone (6PPD-Q), which are the quinone compounds transformed from 6PPD that undergo chemical reactions under the action of light and ozone and were extremely toxic to aquatic organisms, while the median lethal dose (LC50) for coho salmon was 95 ng/L [10]. And it was also toxic to other aquatic organisms [11,12,13,14], as shown in Table S2. 6PPD and 6PPD-Q enter the aquatic environment through runoff from rainwater through TWPs [3,15,16,17]. They were widely present in surface water [18], lake sediments [19], indoor and outdoor dust [20], and drinking water [21]. A nationwide survey conducted by the Chinese scholars showed that the annual river runoff of 6PPD and 6PPD-Q was approximately 3.77 tons per year and 87.3 tons per year, respectively [22]. In addition, 6PPD and 6PPD-Q have been detected in foods such as freshwater fish [23] and leafy vegetables [24,25]. They can enter the human body through the food and have been found in human urine [26], breast milk [27,28], blood [29], and cerebrospinal fluid [30], posing a potential threat to ecosystems and human health. Therefore, 6PPD-Q has received widespread attention from the scientific community [31].

Therefore, many countries, regions, or organizations have taken strict control measures as follows. Washington State Act No. 5931 requires a comprehensive review and control of motor vehicle tires containing 6PPD, and has issued a plan to develop alternative substances for 6PPD. The American Tire Manufacturers Association has released *N*-(1,4-Dimethylamyl)-*N*’-phenyl-*p*-phenylenediamine (7PPD), *N*-Isopropyl-*N*’-phenyl-*p*-phenylenediamine (IPPD), *N*, *N*’-bis(1,4-dimethylpentyl)-*p*-phenylenediamine (77PD), and *N*, *N*’-dicyclohexyl-*p*-phenylenediamine (CCPD) compounds as substitutes for 6PPD. The U.S. Department of Toxic Substances Control (DTSC) has included 6PPD in the list of priority monitored products and requires tire manufacturers selling tires to evaluate safer alternatives in California [9]. The European Union is taking measures to restrict the use of 6PPD in tires, and Austria and the Netherlands will propose a restriction proposal for 6PPD in tires. The U.S. Environmental Protection Agency (U.S.EPA) has listed 6PPD-Q as a priority pollutant for monitoring and requires a comprehensive assessment of the environmental exposure risk of 6PPD-Q [32].

Currently, scholars have conducted in-depth studies on the distribution, migration, and transformation laws of 6PPD and 6PPD-Q in environmental media, as well as their toxicology on aquatic animals and plants, the results of which have been gradually made public, providing basic support for the formulation of relevant regulations. In October 2022, the German Federal Institute for Risk Assessment (BfR) published a standard method for the migration of 6PPD, stipulating that the migration of 6PPD in food or food simulants must not exceed 0.3 mg/L, while China has not established a corresponding standard limit yet. Formulating a standard limit requires accurate measurement of the content and distribution of 6PPD and 6PPD-Q in various matrices, which requires extensive monitoring data. Therefore, there is an urgent need to develop a comprehensive, systematic, and precise detection technology. However, the qualitative and quantitative analysis of these compounds still faces numerous technical challenges. To address these technical challenges in the analysis of PPDs and their transformation products, researchers have developed various sample pretreatment methods and detection technologies. This article systematically reviewed the toxicity and human hazard risks of 6PPD and 6PPD-Q, comparing the advantages and disadvantages of sample pretreatment methods and detection technologies for 6PPD and 6PPD-Q in various food matrices, aiming to provide a scientific basis for food safety risk assessment.

## 2. Toxicity and Human Health Risks of 6PPD and 6PPD-Q

Early studies by Chinese researchers demonstrated that 6PPD exerts toxic effects on human cells. A concentration-dependent increase in cytotoxicity, with higher 6PPD levels leading to greater inhibition of cell proliferation [33,34]. These findings collectively confirm the ability of 6PPD to inhibit cellular proliferation and trigger programmed cell death, while neither of them assessed the cytotoxic potential of 6PPD-Q. For the past few years, domestic and foreign researchers have further confirmed the cytotoxicity of 6PPD-Qthrough in vitro cell-based experiments. Nair et al. [35] demonstrated that 6PPD-Q exhibited significant cytotoxic effects after exposing silver frog CSE-119 cells to the compound for 48 h, with a half-maximal effective concentration (EC50) of 16.98 µg/L. Studies by the cell-based simulation in vitro, Tang et al. [36] revealed that 6PPD and 6PPD-Q stimulate endometrial cell proliferation via the estrogen receptor-α (ERα) and G protein-coupled estrogen receptor (GPER) signaling pathways, promote cell migration through ERα/GPER-mediated regulation of epithelial–mesenchymal transition and inflammatory responses, and induce endometrial cell dysfunction. Concerning human hepatocellular carcinoma (HepG2) cell experiments in vitro, Guo et al. [37] found that 6PPD and 6PPD-Q have potential hepatotoxic exposure risks. Zhang et al. [38] conducted vitro experiments, and the multispectral techniques and computational simulations demonstrated that 6PPD-Q exerts a competitive inhibitory effect on acetylcholinesterase (AChE) activity, with a kinetic parameter (*K_m_*) ranging value from 0.058 to 0.088 mM. In the presence of 6PPD-Q, the AChE structure becomes destabilized and looser, thereby hindering substrate binding and impairing enzymatic activity, revealing that 6PPD-Q may possess potential neurotoxic effects. Li et al. [39] integrated network toxicology and molecular docking approaches to elucidate the interaction patterns between 6PPD, 6PPD-Q, and the key target cytochrome c, somatic (CYCS). Both compounds were found to impair the mitochondrial electron transport chain, disrupt the apoptotic pathway, and activate the nuclear transcription factor κB (NF-κB) and Janus kinase/signal transducer and activator of transcription (JAK-STAT) signaling pathways, thereby triggering inflammatory cascade reactions and increasing the risk of respiratory inflammation, which possesses respiratory toxicity. By treating mammalian cells and unicellular algae with 6PPD-Q, the results showed that 6PPD-Q can react with deoxyguanosine to form isomer-specific 3-hydroxy-1, *N*^2^-6PPD-ethylidene-2′-deoxyguanosine (6PPDQ-dG). 6PPDQ-dG was also detected in the genomic DNA of cells and this 6PPDQ-DNA adduct can damage DNA and has genotoxicity [40].

Li et al. [41] conducted transcriptomic, differential gene expression, and network toxicology analyses on the livers of Kunming mice exposed to 4 mg/kg of 6PPD-Q, the result of which indicated that 6PPD-Q primarily induces hepatotoxicity through apoptosis, inflammation, and lipid metabolic disturbances. Ma et al. [42] subjected male C57BL/6 mice to prolonged exposure to 4 mg/kg of 6PPD-Q, and revealed significant cognitive impairments in the treated mice by behavioral assessments. Biochemical analyses demonstrated elevated levels of reactive oxygen species, increased apoptosis, and disruption of the blood–brain barrier (BBB). Furthermore, heightened levels of pro-inflammatory cytokines, including TNF-α, IL-6, and IL-1β, along with microglial activation, were observed, indicating that 6PPD-Q induces a robust neuroinflammatory response. These findings confirm the toxicity of 6PPD-Q to a mammalian nervous system.

The studies have demonstrated through animal experiments that 6PPD and 6PPD-Q exhibit cytotoxicity, hepatotoxicity, neurotoxicity, and genotoxicity. Furthermore, 6PPD-Q exhibit significant aquatic toxicity, inducing acute toxicity in certain salmonid species and causing developmental toxic effects. In 2020, Tian et al. [8] conducted a pioneering study demonstrating a direct association between 6PPD-Q exposure and acute mortality in coho salmon (*Oncorhynchus kisutch*). Subsequently, in 2022, the same research group determined the median lethal concentration (LC50) of 6PPD-Q for coho salmon to be 95 ng/L [10]. Furthermore, Greer et al. [43] employed whole-transcriptome sequencing to investigate the underlying mechanisms, suggesting that BBB disruption may be a key contributor to 6PPD-Q-induced mortality in salmonids, and proposed the bioaccumulation possibility of 6PPD-Q. Following these findings, extensive studies have been undertaken on the aquatic toxicity of 6PPD-Q, as summarized in Table S2. The study by Brinkmann et al. [14] was the first to demonstrate the acute toxicity of 6PPD-Q to rainbow trout (*Oncorhynchus mykiss*) and brook trout (*Salvelinus fontinalis*), which are species of commercial, aquacultural, and ecological significance, and highlight the need for comprehensive assessment of both acute and sub-lethal effects across diverse fish species.

Epidemiological evidence suggests potential health risks to humans associated with 6PPD-Q. Zhang et al. [44] collected dust samples from roads (*n* = 40), homes (*n* = 91), and kindergartens (*n* = 52) in Guiyu (the e-waste-exposed group) and Haojiang (the reference group) between 2019 and 2021, the content of 6PPD-Q in the collected sample was determined. The findings revealed higher concentrations of 6PPD-Q in children exposed to electronic waste compared to unexposed controls. The estimated daily intake of 6PPD-Q via ingestion was approximately fivefold greater than that via inhalation, and higher daily intake levels were significantly associated with lower body mass index (BMI) as well as increased frequencies of influenza and diarrhea among children. In a separate study, Lv et al. [45] analyzed urinary concentrations of 6PPD and 6PPD-Q in 329 healthy controls and 367 colorectal cancer patients from Quzhou, China. The median urinary 6PPD-Q concentration in colorectal cancer patients (0.94 μg/g creatinine) was significantly elevated relative to the control group (Mann–Whitney U test, *p* = 0.001). Elevated urinary levels of 6PPD-Q were significantly associated with an increased risk of colorectal cancer, and concurrent exposure to both 6PPD and 6PPD-Q was positively associated with higher disease risk. Furthermore, an analysis of serum and urine samples from indoor and outdoor workers demonstrated that outdoor workers had serum 6PPD (0.54–1.66 μg/L) and 6PPD-Q (0.58–4.04 μg/L) concentrations approximately two- and threefold higher, respectively, than their indoor counterparts. Additionally, 18 biochemical parameters (particularly total bilirubin and indirect bilirubin) were significantly elevated (*p* < 0.05). Statistical analyses revealed significant positive correlations between serum 6PPD-Q levels and immune cell counts, total bilirubin, indirect bilirubin, and triglycerides (*p* < 0.05). Logistic regression analysis showed that for each 1 μg/L increase in serum 6PPD-Q, the risk of human liver disease increased by 2.31 times. Outdoor exposure was associated with increased concentrations of 6PPD-Q in serum, which could potentially influence glucose and lipid metabolism, immune cell regulation, and liver health [46].

## 3. Food Matrix Pretreatment Method

### 3.1. Liquid–Liquid Extraction (LLE) Method

To assess the distribution of 6PPD and 6PPD-Q in leafy vegetables and their implications for human dietary exposure, Sherman et al. [25] employed acetonitrile as the extraction solvent, performing multiple extractions from leafy vegetable samples. The combined extracts were concentrated to 5 mL and filtered through a 0.2 μm nylon membrane prior to analysis. The limits of quantification (LOQs) for 6PPD and 6PPD-Q were established at 0.1 μg/kg and 2.8 μg/kg, respectively, with spiked recovery rates ranging from 104% to 151% and 105% to 125%, demonstrating good accuracy and reliability. To study the distribution and transformation products of 6PPD in the leaves and roots of leafy vegetables, Wang et al. [47] and Castan et al. [48] both utilized acetonitrile as the extraction solvent to extract 6PPD and 6PPD-Q from Chinese cabbage and lettuce. Quantification was performed using the isotope internal standard method, and analytes were determined by the UPLC-MS/MS. These LLE methods provide critical technical support for evaluating human exposure risks associated with these contaminants. The use of acetonitrile enables efficient extraction of 6PPD and 6PPD-Q from plant matrices, while direct injection of extracts minimizes analyte loss during sample preparation. Furthermore, this efficiency is attributable to the solubility of 6PPD and 6PPD-Q in acetonitrile (Table S1). However, LLE requires repeated extractions, substantial volumes of organic solvents, and lacks a matrix cleanup step, limiting its applicability to complex animal-derived food matrices.

### 3.2. Accelerated Solvent Extraction (ASE) Method

Breider et al. [24] used ASE technology to extract 6PPD and 6PPD-Q from leafy vegetables (lettuce, cabbage, spinach), rhizomes (onion, potato, carrot) and melons and fruits (tomato, sweet pepper, zucchini, pumpkin) vegetables. Following two extraction cycles, the combined extracts were concentrated to 1 mL and diluted with a solution of 5% acetonitrile in water containing 0.1% formic acid. The resulting solution was directly analyzed using the UPLC-MS/MS. The LOQ was established at 0.3 µg/kg, with spiked recovery rates ranging from 39% to 121% for 6PPD and 82% to 88% for 6PPD-Q. A key advantage of ASE is its significant reduction in solvent consumption, and the solvent consumption is reduced by more than 90%compared to conventional liquid–liquid extraction methods.

### 3.3. Solid-Phase Extraction (SPE) Method

Solid-phase extraction (SPE) is a widely employed sample preparation method for the separation and enrichment of target analytes. It has been extensively applied in the pretreatment of veterinary drug residues and environmental contaminants in complex food matrices. Although there are currently no published studies on the use of SPE for the pretreatment of 6PPD and 6PPD-Q in food samples, recent research has demonstrated its application in the enrichment and purification of 6PPD-Q in aqueous matrices, including surface water, drinking water, and urban runoff water. But in recent years, the U.S.EPA has incorporated SPE into a draft analytical method for the determination of 6PPD-Q in surface water. In this protocol, 250 mL of the water sample was processed using a Strata-XL cartridge (200 mg, 6 mL), followed by elution with acetonitrile and concentration to 10 mL prior to quantification via isotope internal standard combining with mass spectrometry [49]. Another study filtered 50 mL of runoff water through a 1.2 μm glass fiber filter and subsequently purified and enriched the sample using a hydrophilic–lipophilic balance (HLB) SPE cartridge (60 mg, 3 mL), with elution performed using 3 mL of a methanol–dichloromethane mixture (1:9, *v*/*v*) [50]. Additionally, 10 mL of clarified water sample was processed on an Oasis HLB SPE cartridge (30 mg, 1 mL) and eluted in four fractions totaling 1.6 mL of methanol, achieving recovery rates of 6PPD-Q between 78% and 91% [51]. Marques dos Santos et al. [21] processed 1 L of drinking water using an Oasis HLB cartridge (500 mg, 6 mL), eluting sequentially with 5 mL of methanol and 5 mL of a methanol–methyl tert-butyl ether solution (1:9, *v*/*v*), followed by nitrogen evaporation and reconstitution in 250 μL of methanol prior to injection, reporting spiked recoveries of 80–90%. Despite its effectiveness, conventional SPE involves a labor-intensive procedure that typically includes multiple steps, such as cartridge conditioning, sample loading, washing, and elution, often requiring the use of various solvents. Moreover, the substantial consumption of organic solvents raises concerns regarding environmental sustainability and green chemistry principles. Sometimes the different solvents need to be replaced, and the nitrogen drying step is indispensable, which will take a relatively long period of time for the detection.

### 3.4. QuEChERS Method

This is a sample pretreatment method characterized by rapidity, simplicity, cost-effectiveness, high efficiency, robustness, and safety. The procedure involves an initial extraction step, followed by cleanup of the extract using dispersive solid-phase extraction (d-SPE) [52]. Originally designed for the extraction and purification of pesticide residues in fruits and vegetables, the QuEChERS method has since been widely adopted for the analysis of various analytes across diverse matrices due to its operational simplicity and speed, thereby significantly enhancing analytical throughput [53]. For instance, in honey samples with high sugar content, 6PPD and 6PPD-Q were extracted using the deionized water and acetonitrile, followed by salting-out with sodium chloride. The resulting supernatant was purified using 30 mg primary secondary amine (PSA) and 100 mg anhydrous magnesium sulfate, after which the cleaned extract was directly analyzed by UPLC-MS/MS [23]. In the case of fish tissue samples with complex matrices, following extraction and salting-out with sodium chloride, a mixed d-SPE sorbent comprising C18, PSA, and anhydrous magnesium sulfate was employed to effectively remove interfering substances such as proteins and lipids, thereby substantially reducing sample processing time. Recoveries of 6PPD in honey and fish tissue were from 85.4% to 90.5% and 73.3% to 102.7%, respectively, while those of 6PPD-Q ranged from 95.3% to 97.5% and 100.1% to 108.3%, with LOQs between 0.00043 and 0.001 mg/kg [23]. For plant-derived matrices, a similar mixture of C18, PSA, and anhydrous magnesium sulfate has also proven effective for the extraction and purification of 6PPD and 6PPD-Q [31,54]. Nevertheless, a key limitation of the QuEChERS method is its suboptimal purification performance for samples with low moisture or high fat content, which can lead to reduced extraction efficiency and significant analyte loss during cleanup. Furthermore, another major drawback is the inability to change solvents between the extraction and pre-concentration stages, limiting its flexibility in downstream analysis [55].

### 3.5. Pass-Through SPE Column Method

In the analysis of complex matrices, conventional purification materials have increasingly revealed limitations such as low specificity and inadequate purification efficiency, prompting ongoing development of novel sorbents. By integrating these advanced materials with the solid-phase extraction (SPE) method, pass-through SPE column methods have emerged as a promising alternative. For instance, Oasis PRiME HLB and EMR-Lipid columns are now widely applied in the cleanup of residues and contaminants. The Oasis PRiME HLB column offers high sample loading capacity, operational simplicity, and high extract purity [56]. The EMR-Lipid column contains a unique adsorbent that selectively removes lipids from complex matrices without compromising analyte recovery [57,58]. This streamlined, single-step SPE approach eliminates the need for column activation and allows direct loading of small sample volumes, significantly reducing organic solvent consumption and aligning with green chemistry principles. Yuan et al. [59] extracted 6PPD from milk samples using acidified acetonitrile (5% formic acid, *v*/*v*) followed by cleanup with a Captiva EMR-Lipid column, achieving the limit of detection (LOD) of 7.1 μg/L and spiked recoveries ranging from 97.73% to 99.87%. In the determination of 6PPD-Q in fish tissues, Moody et al. [60] developed an ASE method, coupled with EMR-Lipid column purification. The method demonstrated absolute recovery of the isotope-labeled internal standard between 80% and 96%, the LOQ was below 0.7 ng/g, a relative standard deviation of less than 9%, and excellent repeatability, thereby providing a reliable analytical method for toxicokinetic studies of 6PPD-Q.

### 3.6. Multi-Plug-Filtration-Cleanup (m-PFC) Method

The m-PFC method represents advancement over the QuEChERS method, incorporating a novel multi-level composite material engineered through nanotechnology. This composite, consisting of functionalized multi-walled carbon nanotubes and lipophilic adsorbents, is integrated into a solid-phase extraction (SPE) column format. It effectively removes matrix interferences such as pigments, lipids, certain sugars, and sterols, operating on a selective adsorption principle that retains impurities while allowing target analytes to pass through unretained. The purification process is achieved by passing the sample extract through the sorbent bed under plunger pressure or vacuum suction, enabling rapid cleanup within seconds. Compared with the SPE process, the m-PFC procedure can be performed in a few seconds without the leaching and elution steps [61,62]. Compared with the QuEChERS approach, the m-PFC also mitigates the issue of graphitized carbon black (GCB) adsorbing planar-structured compounds, which often leads to analyte loss. Meanwhile, it eliminates the need for vortex mixing and centrifugation during the cleanup procedure. As to the immaturity of the analytical methods for 6PPD and 6PPD-Q in aquatic products, Zhang et al. [63] developed an m-PFC-based purification protocol for the rapid determination of multiple phenylenediamines, including 6PPD and 6PPD-Q. The method employed the 0.1% sodium hydroxide-acetonitrile solution as the extraction solvent, combined with salting-out-assisted extraction and m-PFC cleanup, thereby enhancing purification efficiency and achieving satisfactory recoveries ranging from 62.1% to 115%. The LOD of the method ranged from 0.00300 to 0.400 μg/kg. Table S3 summarizes the comparison of pretreatment methods for 6PPD and 6PPD-Q across various food matrices.

## 4. Analysis and Detection Techniques

### 4.1. Ultra-High-Performance Liquid Chromatography–Tandem Mass Spectrometry (UPLC-MS/MS) Method

Ultra-high-performance liquid chromatography–tandem mass spectrometry (UPLC-MS/MS) enables direct analysis of most pollutants without the need for derivatization. Owing to its high sensitivity and accurate, reliable results, it has become a predominant technique in multi-residue analysis methods [64]. Among currently reported analytical approaches, UPLC-MS/MS has emerged as the mainstream method for the detection of 6PPD and 6PPD-Q, and is widely employed in food safety and toxicological research by researchers worldwide.

Food safety: UPLC-MS/MS method offers significant advantages for food safety detection, including high sensitivity and broad applicability. For instance, a C18 reversed-phase column (e.g., Waters Acquity UPLC BEH C18) was employed with acetonitrile and 0.1% aqueous formic acid as the mobile phase under gradient elution conditions. Detection was performed in electrospray positive ion mode (ESI^+^) using multiple reaction monitoring (MRM), with precursor-to-product ion transitions of *m*/*z* 267.2 > 134.1 and *m*/*z* 279.1 > 171.1 selected as the quantitative ion pairs for 6PPD and 6PPD-Q, respectively. When combined with QuEChERS sample pretreatment, the method LOQs are 0.1 μg/kg for 6PPD in honey and 0.43 μg/kg in fish, while those for 6PPD-Q are 0.3 μg/kg in honey and 1.0 μg/kg in fish [23]. Under similar chromatographic and mobile phase conditions, another study applied accelerated solvent extraction (ASE) for the extraction of 6PPD-Q from fish samples, followed by cleanup using an EMR-Lipid column and analysis by the UPLC-MS/MS, achieving LOQs of 0.37–0.67 μg/kg. The LOQs were more than 1.5-fold lower than those reported in the previous study, and have demonstrated enhanced sensitivity [60].

Biological toxicity study: Tian et al. [8] employed ultra-high-performance liquid chromatography–high-resolution tandem mass spectrometry (UPLC-HRMS/MS) to identify the primary toxicant responsible for acute mortality in coho salmon exposed to tire wear particle (TWP) leachates. UPLC-QTOF-MS analysis confirmed that the toxicant was 6PPD-Q, an oxidation transformation product of 6PPD. This marked the first report identifying 6PPD-Q as a causative agent of acute death in coho salmon. In 2022, Tian et al. [10] quantified 6PPD-Q in aquatic habitats of coho salmon using high-purity commercial standards and an isotope-labeled internal standard method coupled with UPLC-MS/MS. The LOQ ofthe method was below 10 ng/L. Concurrently, the acute toxicity assays were conducted, yielding an LC50 value of 95 ng/L for 6PPD-Q toward coho salmon, and thereby confirmed its significant lethality to aquatic organisms. Subsequently, researchers worldwide have conducted extensive studies on the biotoxicity of 6PPD-Q, utilizing UPLC-MS/MS for the determination of trace levels of 6PPD and 6PPD-Q in various aquatic matrices, as summarized in Table S2.

### 4.2. Gas Chromatography–Mass Spectrometry (GC-MS/MS) Method

Kuo et al. [65] established a method for extraction and quantitative analysis of 6PPD-Q in complex tissues from shellfish, finfish, and marine mammals. The method is based on gas chromatography–tandem mass spectrometry (GC-MS/MS). A total of 45 mL dichloromethane was used as the extraction solvent. Following the ASE, the extract was purified using a silica–alumina column and eluted with 50 mL of a methanol–dichloromethane mixture (15:85, *v*/*v*). The eluate was evaporated under a stream of nitrogen in avacuum, and then reconstituted in 250 μL of dichloromethane. Further purification and concentration were achieved by gel permeation chromatography (GPC), after which the sample was re-dissolved in 200 μL of isooctane. IPPD-Q-d5 was added as an instrumental internal standard prior to analysis. The method was applied to real-world samples, including filets and whole organisms such as mussels, English sole, Chinook salmon, pink salmon, juvenile silver salmon, and steelhead. The LOQ for 6PPD-Q ranged from 0.03 to 0.12 ng/g, and the recovery of the sample preparation internal standard (6PPD-Q-d5) was between 60% and 100% [65]. These results demonstrate that the method is suitable for the analysis of 6PPD-Q in complex biological matrices, including fish, shellfish, and marine mammals. The advantages of GC-MS/MS include the use of an ion source distinct from that of liquid chromatography–tandem mass spectrometry (LC-MS/MS), which typically employs electrospray ionization (ESI), thereby minimizing ion suppression effects. Additionally, GC-MS/MS instruments are widely available and generally more cost-effective than LC-MS/MS systems, making them well-suited for the analysis of volatile and semi-volatile organic compounds.

### 4.3. High-Resolution Mass Spectrometry (HRMS) Method

High-resolution mass spectrometry (HRMS) enables analysis of analytes at elevated resolution, providing accurate molecular mass measurements of precursor ions and extensive fragment ion information. It exhibits strong resistance to matrix interference, thereby reducing the risk of false-positive identifications. In the absence of reference standards or established fragmentation patterns, HRMS software (such as Compound Discoverer 3.0, Mass Frontier 8.1, ect.) can predict elemental compositions, assist in structural elucidation, and confirm metabolites derived from parent compounds [64]. This capability offers robust support for the screening of 6PPD and 6PPD-Q in food matrices. HRMS technologies are primarily classified according to mass analyzer principles into quadrupole-Orbitrap (Q-Orbitrap) and quadrupole-time-of-flight (Q-TOF) systems. These are typically coupled with liquid or gas chromatography to leverage both separation efficiency and high mass accuracy, giving rise to platforms such as UPLC-HRMS and GC-HRMS. The identification of 6PPD-Q by Tian et al. through screening of toxicants in tire wear particle (TWP) leachates using UPLC-Q Exactive HF has since led to widespread application of HRMS in studies of biological toxicity mechanisms and food safety assessment. To investigate species-specific toxicity and the metabolic relevance of 6PPD and 6PPD-Q, Grasse et al. [15] employed UPLC-HRMS to preliminarily identify 22 biotransformation products of 6PPD and 12 of 6PPD-Q. Their study revealed that after 96 h of exposure to 6PPD in zebrafish embryos, biotransformation products accounted for 47% of the total peak area (TPA), with 4-hydroxydiphenylamine being the most abundant. Upon exposure to 6PPD-Q, over 95% was biotransformed, predominantly to 6PPD-Q + O + glucuronic acid, which contributed to more than 80% of TPA. Among the identified 6PPD metabolites, the reactive *N*-phenyl-*p*-benzoquinoneimine was found. This study advances understanding of the metabolic fate of 6PPD and 6PPD-Q. *Raphidocelis subcapitata* (*R. subcapitata*), a unicellular green algae, was widely used as a model organism in ecotoxicological studies, particularly for assessing aquatic pollutant toxicity. Yan et al. [66] developed a complementary non-targeted analytical strategy for identifying the phase I and phase II metabolism of 6PPD and 6PPD-Q in freshwater microalga *Raphidocelis subcapitata*, combining a stepwise LC-Q-TOF-MS screening approach, which was based on predicted reaction pathways and sequential metabolite tracing, and with signal subtraction techniques using GC × GC-TOF-MS. The results demonstrated successful identification of 24 metabolites of 6PPD-Q by LC-Q-TOF-MS and 15 6PPD metabolites by LC-Q-TOF-MS and GC × GC-TOF-MS, including 9 previously unreported metabolites. This study revealed, for the first time, that algae can rapidly transform 6PPD and 6PPD-Q via multiple metabolic pathways, with certain metabolites exhibiting higher toxicity than their parent compounds, which poses potential risks to the aquatic food web. The integrated stepwise screening and signal subtraction approach establishes a novel paradigm for metabolite identification in complex environmental matrices [66].

### 4.4. Condensed Phase Membrane Incorporated Mass Spectrometry (CP-MIMS) Method

The CP-MIMS method is an online, in situ analytical technique employed for the analysis and quantification of low-volatility analytes. In this method, the analyte diffuses through a hollow fiber membrane, dissolves into a liquid receiving phase (e.g., methanol), and is subsequently introduced directly into the mass spectrometer for detection [67]. Key advantages of CP-MIMS include minimal sample pretreatment requirements, rapid response, high sensitivity, and suitability for analyzing low-volatility compounds. For instance, Monaghan et al. [68] applied this technique to determine 6PPD-Q in water samples, demonstrating a simple procedure with detection sensitivity below 8 ng/Land the analysis time of approximately 2.5 min per sample. Compared to conventional chromatography–mass spectrometry methods, CP-MIMS eliminates the need for extraction, cleanup, and pre-concentration steps, thereby significantly reducing both time and cost while enhancing analytical throughput.

### 4.5. Rapid Detection Method

Recently, Li et al. [69] developed a rapid detection kit for determining 6PPD in tire rubber. This method is based on the high oxidation potential of 6PPD, which, in the presence of potassium persulfate (K_2_S_2_O_8_), rapidly loses two electrons and two protons to form a red-colored oxidation product, *N*-1,3-dimethylbutyl-*N*’-phenylquinonediimine (6QDI). Protonated 6QDI exhibits high water solubility, allowing the reaction to proceed efficiently even when the concentration of 6PPD exceeds its aqueous solubility limit (1.88 mg/L). A key advantage of this approach is the color contrast: 6PPD is colorless, whereas 6QDI is distinctly red, enabling visual detection without instrumentation. The method allows on-site analysis, requires minimal energy and reagents, generates little waste, and is safe and user-friendly. It does not necessitate specialized chemical training or sophisticated analytical equipment. By overcoming the limitations of traditional laboratory-based methods, such as long turnaround times and high costs, this technology offers a promising solution for real-time monitoring and regulatory compliance. It holds potential for adaptation to rapid food safety screening, thereby enhancing the efficiency of on-site supervision. The superiority of the different detection methods for 6PPD and 6PPD-Q was shown in the Table S4.

### 4.6. Other Detection Methods

Choi et al. [70] developed a high-performance liquid chromatography–ultraviolet detection (HPLC-UV) method for the quantification of 6PPD in mouse liver samples. The maximum absorption wavelength of 6PPD was 290 nm, and the deionized water and acetonitrile (1.5:8.5, *v*/*v*) was used as the mobile phase. The LOD of the method was 0.17 ng/mL. This method demonstrates high sensitivity, simplicity, and absence of matrix interference, making it suitable for routine applications or laboratories with limited resources.

In order to minimize sample pretreatment and concentration steps, and thereby reduce the risk of contamination during laboratory handling, Marques dos Santos et al. [71] employed large-volume direct injection liquid chromatography–tandem mass spectrometry (LVI-LC-MS/MS) method for the determination of 6PPD and 6PPD-Q in bottled water. The injection volume of method was 100 μL. The LOD of 6PPD was 0.1 ng/L, and the LOD of 6PPD-Q was 0.025 ng/L. This approach was simple, rapid, and did not require sample pretreatment or enrichment. However, it relies on highly sensitive mass spectrometric instrumentation and is currently limited to the analysis of relatively clean aqueous samples.

## 5. Challenges and Future Perspectives

In summary, sample pretreatment methods and analytical technologies for 6PPD and its transformation product, 6PPD-Q, have evolved into a multidimensional and complementary framework. UPLC-MS/MS, as a well-established technique for separation and confirmation, offers high sensitivity and robust quantitative performance; in the years to come, it will provide reliable data support for future studies on the environmental distribution, food safety monitoring, and biotoxicity assessment of 6PPD and 6PPD-Q. HRMS has become a mainstream approach for the comprehensive structural characterization of PPDs and their transformation products in complex matrices, offering strong capabilities for high-throughput screening, identification, and confirmation. Techniques such as UPLC-HRMS and GC-HRMS are increasingly applied to investigate potential transformation pathways and products of 6PPD in environmental and biological systems, thereby enhancing understanding of its environmental fate and toxicological behavior, and supporting regulatory agencies in developing science-based risk management strategies. Meanwhile, rapid test kits, with their advantages of rapidity, portability, low cost, and ease of use, are being advanced through the integration of novel materials to improve the sensitivity and selectivity of the methods, further expanding their applicability for on-site detection of 6PPD.

However, first, the biological toxicity data is still insufficient. The conversion products of 6PPDare more than 6PPD-Q, whose toxic effects of transformation products exhibit greater than 6PPD, while current knowledge regarding the absorption, distribution, metabolism, and excretion pathways of 6PPD and its quinone derivatives (6PPD-Qs) in biological systems is limited, requiring further research to elucidate the potential health risks and underlying toxicological mechanisms associated with the biotransformation and degradation products of these chemicals in vivo. Second, existing detection technologies face multiple technical challenges: (1) Trace-level detection and matrix interference: The concentrations of 6PPD and 6PPD-Q in food matrices are typically extremely low (often below 1 μg/kg) and prone to interference from co-existing components such as lipids and proteins. The enrichment efficiency of current sorbent materials is inadequate, necessitating the development of novel adsorbents with enhanced specificity. (2) Complexity of transformation products: In environmental systems, 6PPD can generate various derivatives, including 6PPD-imine and *N*-formyl-6PPD, for which toxicity data are largely unavailable. High-resolution mass spectrometry combined with in silico toxicological prediction has emerged as a promising approach to address this gap. (3) Accuracy of on-site detection: Portable analytical devices generally exhibit lower quantitative accuracy compared to laboratory-based instruments, limiting their reliability for precise field measurements.

Future development trends include the integration of multi-omics technologies, such as metabolomics based on high-resolution mass spectrometry and mass spectrometry imaging, to enable comprehensive tracking of the absorption, distribution, and metabolic pathways of 6PPD-Q in food matrices and biological tissues. Leveraging the high precision and quantitative capabilities of UPLC-MS/MS, ongoing efforts focus on conducting risk assessments of 6PPD and 6PPD-Q, in foodstuffs and evaluating human exposure levels. By integrating data on food consumption patterns, estimated daily intake, and associated health risks, researchers aim to establish standardized analytical methods and science-based residue limits for 6PPD and 6PPD-Q in foods, with particular emphasis on aquatic products.

## 6. Conclusions

The widespread use of the antioxidant 6PPD results in its rapid dispersion across environmental media and its transformation into more toxic derivatives like 6PPD-Q.The animal experiments have demonstrated 6PPD-Q exhibits cytotoxicity, hepatotoxicity, neurotoxicity, and genotoxicity. Unfortunately, 6PPD and 6PPD-Q have been detected in human biological matrices, raising significant concerns for human health. In addition to drinking water, dietary exposure represents a major pathway for human exposure to 6PPD-Q.An increasing number of studies have reported the presence of 6PPD and 6PPD-Q in food, a finding that warrants serious concern. Therefore, rapid identification and accurate quantification of 6PPD and 6PPD-Q in food are essential.

At present, the pretreatment methods of 6PPD and 6PPD-Q in food matrices have LLE, ASE, SPE, QuEChERS, pass-through SPE, and m-PFC methods. LLE is simple and cost-effective but has limited applicability; furthermore, the extensive use of organic solvents poses health hazards to laboratory personnel and contributes to environmental pollution. SPE and ASE offer high enrichment efficiency and recovery rates yet require specialized instrumentation, thereby increasing operational costs. QuEChERS, pass-through SPE, and m-PFC are rapid and convenient approaches, but their selectivity is highly dependent on the choice of adsorbent material tailored to specific sample matrices, limiting broad applicability. Therefore, future advancements should focus on integrating innovative materials, such as covalent organic frameworks (COFs) and immunomagnetic beads, into sample preparation workflows to improve enrichment efficiency and specificity. UPLC-MS/MS enables precise quantification of trace-level analytes, while HRMS offers distinct advantages in elucidating environmental transformation pathways and enabling non-targeted screening. They have become established as mainstream techniques of 6PPD and 6PPD-Q. There is a growing need to develop more sensitive, accurate, and efficient analytical methods to mitigate analyte loss during sample pretreatment and enhance overall method reliability.

Furthermore, although analytical methods for the detection of 6PPD and 6PPD-Q in food have been reported, the range of tested food matrices remains limited, primarily restricted to aquatic products (including freshwater and marine fish) and leafy vegetables. There is a need to develop more high-throughput, automation, and analytical methods for the determination of 6PPD and 6PPD-Q in a broader range of food matrices to enable comprehensive assessment of health risks associated with dietary exposure. It will provide a scientific basis for establishing maximum residue limits in food.

## Data Availability

No new data were created or analyzed in this study. Data sharing is not applicable to this article.

## Supplementary Materials

The following supporting information can be downloaded at: https://www.mdpi.com/article/10.3390/toxics13121076/s1, Table S1: Structural formulas and physicochemical properties of 6PPD and 6PPD-Q; Table S2: The toxicity of 6PPD and 6PPD-Q on the different aquatic organisms; Table S3: Comparison of pretreatment methods for 6PPD and 6PPD-Q in different food matrices; Table S4: The superiority of the different detection methods for 6PPD and 6PPD-Q.
