# Supplementary material for: Advances in Analytical Determination Methods and Toxicity and Health Risk Assessment of 6PPD and Its Transformation Products in Food
Source: Toxics. 2025 Dec 14;13(12):1076. doi: 10.3390/toxics13121076 (PMC12737381; doi:10.3390/toxics13121076)
Supplement: Supplementary file 1 [file toxics-13-01076-s001.zip › Table S1 Structural formulas and physicochemical properties of 6PPD and 6PPD-Q-manuscript.v7 - proofreading.pdf]

Table S1 Structural formulas and physicochemical properties of 6PPD and 6PPD-Q

Compound	Abbreviation	CAS	Structure	Mr/ g/mol	log K_{oc}	log K_{ow}/ log P	Solubility	Boiling point /℃
N-(1,3-Dimethylbutyl)-N'-phenyl -p-phenylenediamine	6PPD	793-24-8	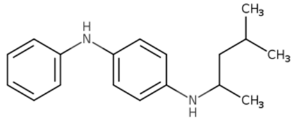	268.19	6.72	5.4	Soluble in Acetonitrile	370
N-(1,3-Dimethylbutyl)-N'-phenyl -p-phenylenediamine-quinone	6PPD-Q	2754428-18-5	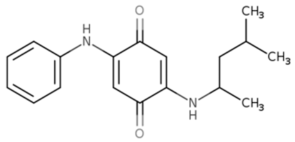	298.17	-	4.1	Soluble in Acetonitrile	442
