# Supplementary material for: Advances in Analytical Determination Methods and Toxicity and Health Risk Assessment of 6PPD and Its Transformation Products in Food
Source: Toxics. 2025 Dec 14;13(12):1076. doi: 10.3390/toxics13121076 (PMC12737381; doi:10.3390/toxics13121076)
Supplement: Supplementary file 1 [file toxics-13-01076-s001.zip › Table S2 the toxicity of 6PPD and 6PPD-Q on the different aquatic organisms-Toxics-manuscript.v7 - proofreading.pdf]

Table S2 The toxicity of 6PPD and 6PPD-Q on the different aquatic organisms

Name	Analyte	Experimental Conditions	Key Findings	Instrumental technique	References
Coho salmon (*Oncorhynchus kisutch*)	6PPD-Q	20 μg/L nominal concentrations，exposure time less than 5 hours	Induced rapid mortality，LC50 value of 0.8 ± 0.16 μg/L	UPLC-HRMS/ UPLC-MS/MS	[8]
Juvenile Coho salmon (*Oncorhynchus kisutch*)	6PPD-Q	Exposure concentration range was from 0.16 to 4.0 μg/L	Mortality rates were 100% at 0.8, 1.8, and 4.0 μg/L, while four of five coho salmon died at 0.16 and 0.36 μg/L; 100% mortality at 0.2 μg/L 6PPD-Q, 67% at 0.1 μg/L, and 0% at 0.02, 0.04, and 0.06 μg/L. LC50 value of 95 ng/L	Isotopic internal standard combined with UPLC-MS/MS	[10]
Lake Trout (*Salvelinus namaycush*)	6PPD-Q	Alevins exposed: the exposure concentration range was 0.22 to 13.5 μg/L, and the duration was 45 days. Acute study: the fish larvae were exposed to the external substances at concentrations of 0.1, 0.3, 0.9, and 2.7 μg/L, with the exposure time being 96 hours.	The young fish showed deformities, and unique blood accumulations were observed in their tail fins and eyes. Alevins exposed from hatch until 45 days posthatch to time-weighted average 6PPD-Q concentrations ranging from 0.22 to 13.5 μg/L exhibited a 45 day median lethal dose (LC50) of 0.39 μg/L；the acute study with exogenously feeding lake trout fry determined a 96 h LC50 of 0.50 μg/L.	UPLC-HRMS	[11]
Juvenile rainbow trout (*Oncorhynchus mykiss*)	6PPD-Q	Exposure concentration was 0.59 μg/L, and the exposure time was 48 h	The measurement of oxygen consumption rate revealed impaired oxygen utilization, reduced oxygen transport in tissues, passive ventricular filling, increased cardiac output, and prolonged PR interval, indicating cardiac stimulation and significant impacts on the cardiovascular and metabolic systems.	Isotopic internal standard combined with UPHLC- HRMS	[12]
Zebrafish (*Danio rerio*)	6PPD<br>6PPD-Q	Exposure concentration was 2 μg/L, 4 μg/L and 8 μg/L, and the exposure time was 28 days	Dysregulation of lipid and carbohydrate metabolic pathways is associated with downregulation of peroxisome proliferator-activated receptor gamma (PPARγ) expression and elevated expression of pro-inflammatory cytokines (TNF-α and IL-6), indicating a potential risk of hepatotoxicity.	LC-MS/MS	[13]
Brook trout (*Salvelinus*	6PPD-Q	Exposure concentration was 0 μg/L, 0.02μg/L, 0.2 μg/L , 2 μg/L, 6 μg/L and	Both brook trout and rainbow trout exhibited 100% mortality within 4 hours of exposure at concentrations of 2 μg/L and 6 μg/L.	Isotopic internal standard combined	[14]

fontinalis), Rainbow trout (*Oncorhynchus mykiss*)		20 μg/L.	The 24 h LC50 for brook trout was 0.59 μg/L, while the 96 h LC50 for rainbow trout was 1.00 μg/L.	with UPHLC- HRMS	
Coho Salmon (*Onchorychus kisutch*)	6PPD-Q	Coho salmon embryos were exposed to three concentrations (0.1, 1, and 10 μg/L) twice weekly, with each exposure consisting of a continuous 24 h period, and the exposure regimen was maintained throughout the embryonic development until hatching.	Acute mortality was not elicited in developing coho salmon embryos, however, growth was inhibited. Following hatching, the risk of mortality increased. Molecularly, 6PPD-quinone induced dose-dependent effects that implicated broad dysregulation of genomic pathways governing cell−cell contacts and endothelial permeability, implicating blood-brain barrier disruption as a potential pathway for toxicity.	Isotopic internal standard combined with UPLC-MS/MS	[43]
