# Supplementary material for: Advances in Analytical Determination Methods and Toxicity and Health Risk Assessment of 6PPD and Its Transformation Products in Food
Source: Toxics. 2025 Dec 14;13(12):1076. doi: 10.3390/toxics13121076 (PMC12737381; doi:10.3390/toxics13121076)
Supplement: Supplementary file 1 [file toxics-13-01076-s001.zip › Table S3 Comparison of pretreatment methods for 6PPD and 6PPD-Q in different food matrix-manuscript.v7 - proofreading.pdf]

Table S3. Comparison of pretreatment methods for 6PPD and 6PPD-Q in different food matrix

Food matrix	Extraction solvent	Purification	Pretreatment processes	Recovery (%)	LOQ/ ng/g	Disadvantages	Advantages	References
Leafy vegetables	Acetonitrile	LLE	The sample was vortexed for 3 min. After centrifugation, 20 mL of the supernatant was collected, and the residue was sequentially extracted twice with 4 mL of extraction solvent. The extracts were combined and filtered through a 0.2 μm nylon membrane. The filtrate was then concentrated to 5 mL under reduced pressure using a rotary evaporator.	104%-151% (6PPD) 105%-125% (6PPD-Q)	0.1 (6PPD) 2.8 (6PPD-Q)	Requires a large amount of organic solvents, results in environmental pollution, time-consuming, and lacks automation capability	Efficient separation, Simple to operate and low in cost	[25]
Fruit, root and leafy vegetables	Acetonitrile	ASE	Extraction procedure: solvent temperature at 80 °C, two extraction cycles, extraction time of 5 min, rinse volume set at 80%, purge time of 60 s. The extract was concentrated to 1 mL and then diluted twofold with a 5% acetonitrile aqueous solution containing 0.1% formic acid. The diluted solution was filtered through a 0.45 μm nylon membrane prior to injection.	39%-121% (6PPD) 82%-88% (6PPD-Q)	0.3	High costs and a significant risk of thermal degradation	Rapid, minimal organic solvent, automation	[24]

Finfish, shellfish, and marine mammal tissues	Dichloromethane	ASE+ GPC	Following ASE extraction, the extract was subjected to aluminum-silica column chromatography using methanol/dichloromethane (15:85, v/v) as the eluent. The collected fraction was concentrated to near dryness under a stream of nitrogen and reconstituted in 250 μL of dichloromethane, followed by purification using GPC purification method.	91%-107% (6PPD-Q)	0.03-0.12 (6PPD-Q)	High costs, complex optimization process	Minimal organic solvent, automation, High extraction efficiency and thorough purification	[65]
Honey	Deionized water and acetonitrile	QuEChERS	The sample was dispersed in 2 mL of ultrapure water and extracted with 10 mL of acetonitrile. Salting-out was achieved by adding sodium chloride, followed by purification using 30 mg of PSA and 100 mg of anhydrous magnesium sulfate.	85.4%-95.7% (6PPD) 95.3%-97.5% (6PPD-Q)	0.1 (6PPD) 1.0 (6PPD-Q)	Select the appropriate adsorbent based on the nature of the analyte, and the limitations.	Rapidity, simplicity, cost-effectiveness, high efficiency, robustness, and safety	[23]

Fish	Acetonitrile	QuEChERS	6PPD: the sample was extracted twice with 20 mL of acetonitrile. Following salting-out with sodium chloride, the extract was purified using a mixture of 600 mg C18, 120 mg PSA, and 120 mg anhydrous magnesium sulfate. 6PPD-Q: the sample was extracted using 5 mL of ultrapure water and 10 mL of acetonitrile, shaken for 10 min, centrifuged at 3800 rpm for 5 min, 6 mL of the supernatant was collected. The residue was re-extracted with 10 mL of acetonitrile. The two extracts were combined, concentrated, and purified using 60 mg C18, 60 mg PSA, and 600 mg anhydrous magnesium sulfate. The final extract was concentrated to near dryness, reconstituted in 1 mL of acetonitrile, filtered through a 0.22 µm nylon membrane, and injected for analysis.	73.3%-102.7% (6PPD) 100.1%-108.3% (6PPD-Q)	0.43 (6PPD) 1.0 (6PPD-Q)	Select the appropriate adsorbent based on the nature of the analyte, and the limitations.	Rapidity, simplicity, cost-effectiveness, high efficiency, robustness, and safety	[23]
Milk	Acetonitrile with 5% formic acid solution	EMR-Lipid	The sample was extracted with 10 mL of acetonitrile containing 5% formic acid, followed by centrifugation. Subsequently, 4 mL of the supernatant was transferred and mixed with 1 mL of ultrapure water. Then, 2.5 mL of the resulting solution was loaded onto a Captiva EMR-Lipid cartridge for purification.	97.73%-99.87% (6PPD)	7.1ng/mL (6PPD)	Select the appropriate adsorbent based on the nature of the analyte, and high costs	Simple operation, wide applicability, and high analysis efficiency	[59]

*GPC: gel permeation chrommatograph
