# Supplementary material for: Advances in Analytical Determination Methods and Toxicity and Health Risk Assessment of 6PPD and Its Transformation Products in Food
Source: Toxics. 2025 Dec 14;13(12):1076. doi: 10.3390/toxics13121076 (PMC12737381; doi:10.3390/toxics13121076)
Supplement: Supplementary file 1 [file toxics-13-01076-s001.zip › Table S4 The superiority of the different detection methods for 6PPD and 6PPD-Q-Toxics-manuscript.v7 - proofreading.pdf]

Table S4. The superiority of the different detection methods for 6PPD and 6PPD-Q

Matrix	Major instrumental techniques used	LOD (analytes)	Analysis time	Superiority	References
Smallmouth bass (*Micropterus dolomieu*), and Coho Salmon (*Onchorychus kisutch*)	UPLC-MS/MS	0.37-0.67μg/kg (6PPD-Q)	10 min	High sensitivity and simultaneous multi-component detection	[60]
Lake trout (*Salvelinus namaycush*),blue mussel (*Mytilus edulis*),English sole (*Parophrys vetulus*),Juvenile Coho salmon (*Oncorhynchus kisutch*),Steelhead (*Oncorhynchus mykiss*),and Pink salmon(*Oncorhynchus gorbuscha*) tissues	GC-MS/MS	0.005-0.041μg/kg (6PPD-Q)	> 20 min	Unlikely to exhibit ion suppression or enhancement	[65]
Raphidocelis subcapitata (*R. subcapitata*)	LC-Q/TOF-MS<br>GC×GC-TOFMS	< 0.01 μg/kg (6PPD-Q)	15-25 min	Stepwise screening, signal subtraction with comprehensive two dimensional, a suite of complementary non-targeted strategy to identify potential metabolites	[66]
Water	CP-MIMS	8 ng/L(6PPD-Q)	2.5 min	Rapid analysis with no requirement for sample pre-processing	[68]
Rubber Products	Rapid Testing Kit	- (6PPD)	< 10 min	Low costs, green and user-friendly for environment, without requiring expertise in chemistry	[69]
